# Multichannel Sensorimotor Integration with a Dexterous Artificial Hand

**DOI:** 10.21203/rs.3.rs-2684789/v1

**Published:** 2023-03-16

**Authors:** Moaed A. Abd, Erik D. Engeberg

**Affiliations:** 1 Ocean and Mechanical Engineering Department, Florida Atlantic University, Boca Raton, Florida, FL, USA; 2 Center for Complex Systems and Brain Sciences, Florida Atlantic University, Boca Raton, Florida, FL, USA

**Keywords:** Sensorimotor Integration, Haptic Feedback, Artificial Intelligence, Slip prevention, Neural Network, Amputee, Prosthetic Hand

## Abstract

**Background:**

People use their hands to perform sophisticated tasks like playing a musical instrument by integrating manifold and diverse sensations of touch with motor control strategies. In contrast, prosthetic hands lack the capacity for multichannel haptic feedback and multitasking functionality remains rudimentary. There is a dearth of research exploring the potential of upper limb absent (ULA) people to integrate multiple channels of haptic feedback into dexterous prosthetic hand control strategies.

**Methods:**

In this paper, we designed a novel experimental paradigm for three ULA people and nine additional subjects to investigate their ability to integrate two simultaneously activated channels of context-specific haptic feedback into their dexterous artificial hand control strategies. Artificial neural networks (ANN) were designed for pattern recognition of the array of efferent electromyogram signals that controlled the dexterous artificial hand. ANNs were also used to classify the directions that objects were sliding across two tactile sensor arrays on the index (I) and little (L) fingertips of the robotic hand. The direction of sliding contact at each robotic fingertip was encoded by different stimulation frequencies of wearable vibrotactile actuators for haptic feedback. The subjects were tasked with implementing different control strategies with each finger simultaneously depending upon the perceived directions of sliding contact. This required the 12 subjects to concurrently control individual fingers of the artificial hand by successfully interpreting two channels of simultaneously activated context-specific haptic feedback.

**Results:**

Subjects were able to accomplish this complex feat of multichannel sensorimotor integration with an overall accuracy of 95.53% ± 0.23%. While there was no statistically significant difference in the classification accuracy between ULA people and the other subjects, the ULA people required more time to correctly respond to the simultaneous haptic feedback slip signals, suggesting a higher cognitive load required by the ULA people.

**Conclusion:**

ULA people can integrate multiple channels of simultaneously activated and nuanced haptic feedback with their control of individual fingers of an artificial hand. These findings provide a step toward empowering amputees to multitask with dexterous prosthetic hands, which remains an ongoing challenge.

## I. Introduction

**Fig. 1.**
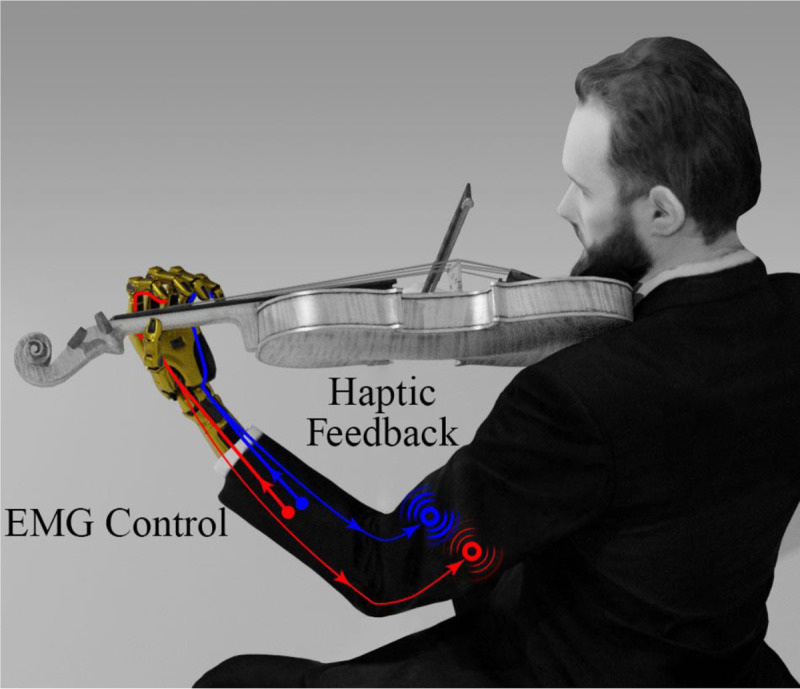
Complex activities such as playing a musical instrument present a great challenge to upper limb amputees. Tasks such as these require accurate slip control of multiple fingertips simultaneously across different surfaces. In this paper, we explore the potential for three amputees and nine non-amputees to simultaneously control the state of sliding contact at two fingertips simultaneously by integrating two channels of variable frequency vibrotactile haptic feedback into their motor control strategies. The rendering of the man in this image was licensed and modified for public display.

The capacity for dexterous control of a prosthetic hand and the sensation of touch are inherently intertwined [1]. Unfortunately, the potential for ULA people to engage in sophisticated activities like playing sports or musical instruments remains elusive because current clinical practice with prosthetic hands affords limited control capabilities, with minimal or no awareness of corresponding touch sensations. Mastery of dexterous tasks requires both the capacity for simultaneous and individual finger control along with context-dependent touch sensations, such as awareness of the direction that fingers are sliding [2] along the strings of a violin [3], for example (Fig. 1). Although this level of control mastery remains largely in the future for amputees, it provides an ambitious goal. To restore potential for multiple independent artificial hand control channels, there are several invasive methods that have been published to enable simultaneous control of multiple degrees of freedom (DOF). Pattern recognition via surgical procedures such as targeted muscle reinnervation [4] or regenerative peripheral neural interfaces [5] can enable simultaneous control of multiple DOFs of a prosthetic arm. Another recent approach detailed 32 intramuscular EMG electrodes that were implanted in the forearms of two amputees to decode individual digit movements with several different deep learning algorithms [6]. There have also been strong advances with neural implants in the peripheral nerves of amputees for direct neural feedback and control [7–9], which could open the gateway to higher control capabilities. While these invasive approaches are truly meritorious, they are not always the best solution for every patient [10].

For this reason, there have been numerous efforts to explore noninvasive methods to increase the human-prosthesis bandwidth [11]. There have been several publications that have demonstrated noninvasive methods for EMG control of a prosthetic wrist [12] while simultaneously opening and closing a prosthetic hand. These efforts detailed multi-layer perceptron [13], linear discriminant analysis [14–16], convolutional neural networks [17], and neuromusculoskeletal models [18] to detect hand motions while wrist pronation/supination or flexion/extension motions were simultaneously performed.

There have also been prior efforts to investigate the ability for simultaneous control of multiple grips or fingers of prosthetic hands independently via noninvasive methods [19]. One major obstacle of this goal is the substantial crosstalk that occurs between muscle recording locations on the forearm [20]. However, there has been progress to decode individual finger movements with ANNs using 32 surface EMG electrodes placed on limb-intact people [21] and 19 electrodes placed on a transradial amputee [22]. Another approach used eight electrode pairs on the forearms of five limb-absent subjects to classify seven different kinds of imagined finger movements with an average classification accuracy of 79% [23]. One paper has detailed the use of different combinations of linear discriminant analysis, principal component analysis, and support vector machines to classify twelve different imagined individual finger movements from six amputees and also combined finger movements from ten limb-intact subjects; high classification accuracies for five distinct finger movements were obtained using six EMG electrode pairs [24].

Regarding noninvasive feedback of haptic sensations, there have been myriad approaches explored in the literature including mechanotactile [25–27], vibrotactile [25, 28, 29], and electrotactile feedback methods [30–32], see [1, 10, 33] for reviews. Particularly relevant to this paper, vibrotactile haptic feedback has been explored due to the light weight and low power requirements of these actuators, which make them conducive to use in wearable prosthetic limbs [34]. Vibrotactile feedback has been of interest to convey haptic feedback for grasped object slip sensations [33] and has been shown to surpass both visual and proportional force haptic feedback in grasped object slip prevention experiments with an artificial hand [35]. The sense of object slippage is particularly important for amputees because they are often unaware of the applied grip force, leading to inadvertently dropping the grasped object [36].

While there have been numerous efforts to prevent slip of grasped objects [37], one commonly overlooked aspect of control is that it is often desirable to permit slip to occur, such as in haptic exploration tasks [38] to manually gather information about the environment [39] with tactile sensor arrays [40] similar to how people use myriad mechanoreceptors in the human fingertips [41]. Fine details of the situation at hand can dictate whether the person would desire to prevent or permit slip [3], such as when handing an object to another person—sliding contact is likely to occur during the handoff [2].

Grasp control challenges compound when multitasking or controlling multiple functions simultaneously. Multichannel haptic feedback arrays show promise to help overcome this difficulty by conveying a broader picture of the hand to the amputee [42] from multiple fingertips simultaneously [43]. However, there is scant research investigating how well ULA people can integrate myriad channels of haptic feedback into their dexterous artificial hand control strategies. The hypothesis of this paper is that amputees can integrate multiple channels of simultaneously activated haptic feedback into their concurrent control of individual fingers of an artificial hand. To test this hypothesis, we developed a novel experimental paradigm where sliding contact occurred in two different directions simultaneously on two fingers of an artificial hand, somewhat like how people slide their natural fingers in different directions simultaneously along the strings of a violin, the keys of a keyboard, or in other tasks of daily life. The sliding sensations of touch from the two robotic fingertips were encoded by the frequency of stimulation based upon the direction of slip at each fingertip and mapped to corresponding wearable vibrotactile actuators. Three ULA people and nine additional subjects were outfitted with an array of EMG electrodes and muscle activation pattern recognition was implemented with an ANN to enable individual and concurrent control of two fingers of the hand. Subjects were trained to recognize the state of sliding contact at both fingertips by the context dependent simultaneously activated haptic feedback and to decide whether to prevent or permit slip of two different objects at the same time.

While there is extant research on EMG pattern recognition for simultaneous multi-DOF control of artificial hands and methods for conveying multiple channels of haptic feedback to ULA people, there is a dearth of research investigating integration of the latter with the former. The novel contribution of this paper is an exploration of ULA people’s capacity for multichannel sensorimotor integration to concurrently control individual fingers of a dexterous artificial hand based on multiple channels of simultaneously activated context-specific haptic feedback.

## II. METHODS

### A. Human Subjects

Twelve human subjects participated in these experiments (six females). Three male subjects had an upper limb amputation or congenital deficiency. The first subject (S1) had a bilateral amputation; transhumeral on the right side, transradial on the left side. He frequently uses several myoelectric and body powered prosthetic devices with his left arm only including the Fillauer Motion Control ETD Hook with a MC wrist rotator (Fillauer, Boulder, USA). The second subject (S2) has a transverse congenital limb deficiency of the left arm slightly beyond the elbow. He often uses a body powered TRS Grip Prehensor (Grip 3 BK). The third subject (S3) has a complex partial left hand amputation hand that was caused by an explosion. He does not use a prosthesis due to the difficulty of fitting a socket to his residual limb. The other nine subjects (S4-S12) had no amputation or congenital limb deficiencies. All participants gave informed written consent under a protocol approved by Florida Atlantic University’s IRB, which was in accordance with the declaration of Helsinki.

### B. Robotic System Hardware

**Fig. 2.**
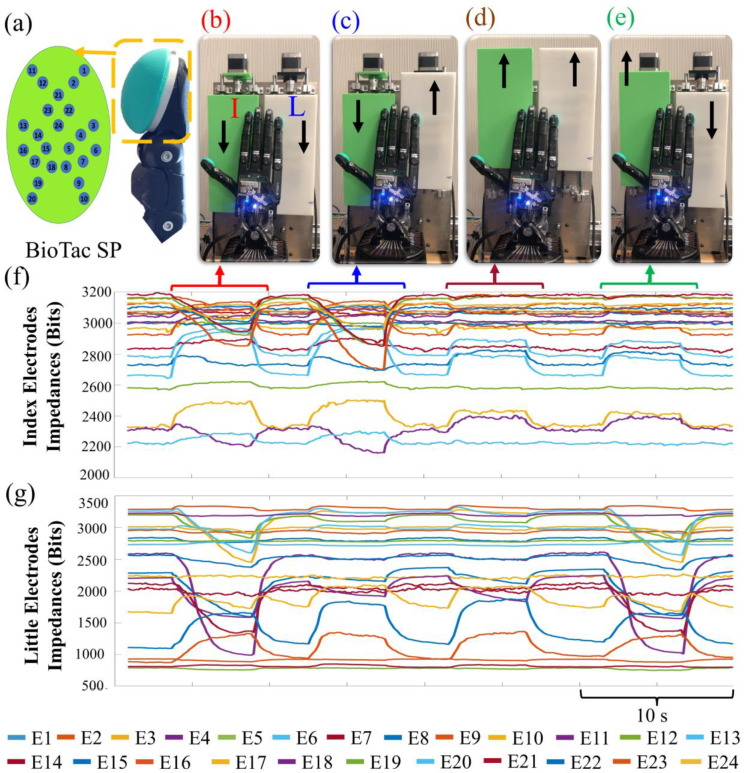
(a) The BioTac SP has 24 sensing electrodes. (b) Photo sequence showing the Shadow Hand in contact with the sliding surfaces. Both the sliding surfaces are slipping down. (c) The index (I) finger surface is slipping down while the little (L) finger surface is slipping up. (d) Both sliding surfaces are slipping up. (e) The I finger surface is slipping up while the L finger surface is slipping down. (f) BioTac SP electrode impedances of the I and (g) L fingers changed characteristically during the different slipping scenarios.

The robotic system used during this experiment includes an E3M Dexterous Shadow Hand (Shadow Robot Company, London, U.K.) fit with BioTac SP tactile sensor arrays (SynTouch, CA) on both the I and L fingertips (Fig. 2(a–e)). The Shadow Hand has 20 tendon-driven DOFs; however, for the purposes of exploring the capacity for simultaneous slip control in this paper, only the metacarpophalangeal joints of the I and L fingers were under control of the users, effectively limiting the DOFs to two. The BioTac SP fingertip tactile arrays are deformable fluid filled sensors that have 24 internal electrodes with impedances that vary as external forces displace the fluid within the cavity (Fig. 2(a)). There is also a hydrophone sensor in each BioTac SP to measure the steady state pressure (P_DC_) within the entire cavity. The dynamic pressure (P_AC_) is also gleaned from P_DC_ with a high pass filter.

Two independently controlled stepper motors (Autonics A2K-M243, TEquipment, NJ) were mounted vertically on an aluminum plate to induce slip either up or down on the BioTacs of the I and L fingers (Fig. 2(b–e)). The stepper motors were rigidly connected to flat rectangular surfaces that were 3D printed with PLA material using an Ultimaker S5 (Ultimaker, Zaltbommel, the Netherlands). Sliding speeds were constant at 0.5 mm/s.

The two vibrotactile stimulators (PN:LS00046, OSEEP Electronics LTD) for haptic feedback from the I and L fingertips were integrated into a stretchable haptic armband made from Dragon Skin-30 (Smooth-On, Inc. Macungie, PA, USA) to deliver information to the subjects about the direction of sliding contact at the fingertips via the frequency of their actuation.

### C. ROS Network Configuration Overview

**Fig. 3.**
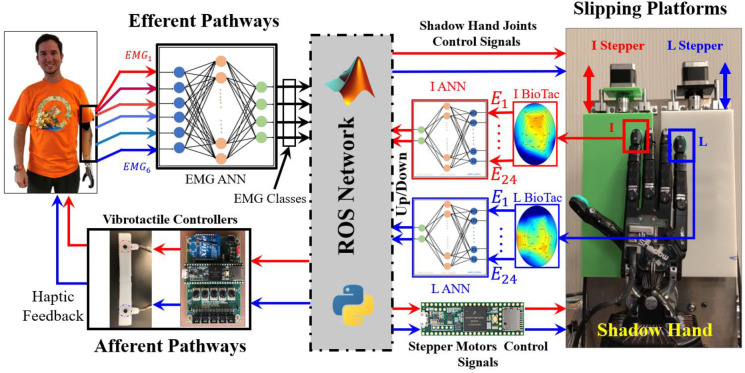
The robotic system configuration. All the system components are interacting with each other through ROS using Python and MATLAB/Simulink. The subject (S2) gave permission for the use of his image.

The system was controlled through a robot operating system (ROS) network comprised of four nodes (Fig. 3). The first three ROS nodes were implemented with the Teensy 3.6 (PJRC.COM, LLC. Sherwood, OR, USA). ROS node 1 was used to control the stepper motors to initiate the slip with respect to the I and L fingers. ROS node 2 was used to control the actuation frequency of the haptic feedback from the vibrotactile stimulators [44] to correspond to either slip in the up or down directions at the I and L fingertips [2]. ROS node 3 was used to sample six EMG signals from the forearms of the human subjects for efferent control of the Shadow Hand to prevent or permit slip with each finger individually or simultaneously. ROS node 4 was deployed in MATLAB/Simulink to implement ANNs to classify the efferent EMG signals and afferent BioTac SP fingertip slip sensations.

### D. Classifying Context-Dependent Slip Sensations From Two Fingertips Simultaneously

To design ANN classifiers to detect the direction of slip on two fingertips simultaneously, data were collected from the BioTac sensors attached to the I and L fingers (Fig. 2 (f, g)). After establishing contact between the fingertips and the sliding surfaces, ten slip trials were induced in the up and down directions. Both ANN classifiers for the fingertips were designed with 24 inputs corresponding to the 24 taxels in each BioTac (Fig. 2a) and 2 outputs for the slip up or slip down classes. Sigmoid and SoftMax activation functions were used for the hidden layer and the output layer respectively. Cross-entropy and confusion matrices were used to evaluate the performance of the ANNs. The networks were trained with scaled conjugate gradient backpropagation using the nprtool in MATLAB. To train and test the ANN, the collected data were divided into 3 categories: training, testing, and validation. The ANNs were trained using 70% of the data and the network was adjusted based on the error generated from this dataset. Network generalization was measured using the 15% validation dataset, and the training was halted when generalization error stopped improving. The 15% testing dataset did not affect the network error and provided a new performance measure that was independent from the training and validation performance measure. See our prior work for more details on the process to classify the direction of sliding contact with ANNs [2].

### E. Haptic Encoding of Task-Relevant Slip Sensations from Two Fingertips Simultaneously

**Fig. 4.**
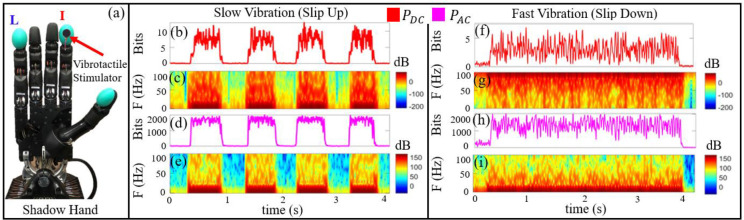
Characterization of the two vibration modes of the vibrotactile stimulators for haptic feedback from the index (I) and little (L) fingers. (a) The BioTac SP on the I finger of the Shadow Hand was used in this experiment to measure the Slow and Fast vibration modes of the vibrotactile stimulators. (b) Steady state pressure (P_DC_) and (c) Spectrogram of the steady state pressure (P_DC_) measured by the BioTac corresponding to the Slow vibration mode. (d) The dynamic pressure signal (P_AC_) and the (e) corresponding spectrogram from the Slow vibration mode. (f) Steady state pressure (P_DC_) and (g) Spectrogram corresponding to the Fast vibration mode. (h) The dynamic pressure signal (P_AC_) and (i) The corresponding spectrogram from the Fast vibration mode.

To encode a directional sense of the sliding motion, each vibrotactile stimulator was toggled on and off at either 1 Hz (Slow) to convey the upward direction or at 200 Hz (Fast) to indicate the downward direction of slip. To objectively quantify the haptic feedback encoding methods from the vibrotactile stimulators, the BioTac SP sensor was utilized as a measurement tool. To that end, the vibrotactile stimulator was connected firmly to the BioTac SP sensor on the I fingertip (Fig. 4(a)). The Slow and Fast vibrotactile activation signals for this test were created in Simulink using the ROS toolbox. The steady state pressure (P_DC_) and dynamic pressure signals (P_AC_) from the BioTac were recorded for both the Slow (Fig. 4(b, d)) and Fast vibration modes (Fig. 4(f, h)).

The time-frequency spectrogram representation for the data was calculated using a 512-point FFT with 0.08 s frame length and a Hanning window with 90% overlap. The time-frequency power distribution data show the frequency components of P_DC_ and P_AC_ for the Slow (slip up; Fig. 4(c, e)) and Fast (slip down; Fig. 4(g, i)) vibrotactile actuation modes. These produced very different activation signatures, which enabled people to clearly distinguish between the different sensations corresponding to upward and downward sliding contact.

### F. Overview of Situationally Aware Multichannel Sensorimotor Integration Experiments for Simultaneous Slip Control

Subjects were trained over the course of approximately two hours to integrate two channels of vibrotactile haptic feedback into their simultaneous control strategies for the I and L fingers. When an object slides along a fingertip, this can be desirable or undesirable depending upon the context. For example, an object sliding upward could be caused when handing an item to another person whereas an object sliding downward could be due to inadvertently dropping an object onto the floor. In the former case, permitting slip would be desirable to hand an object to the other person. In the latter situation, it would be desirable to prevent slip by increasing the grip force. We simulated these scenarios and encoded the vibrotactile actuation frequency from the I and L fingertips to be either Slow (Fig. 4(b–e)) or Fast (Fig. 4(f–i)) to correspond to the slip up or slip down cases, respectively. In the slip up case, subjects were trained to permit slip while in the slip down situation subjects were trained to increase the grip force of the corresponding finger. This necessitated training the subjects to simultaneously interpret the two channels of context dependent haptic feedback to make the corresponding control action with each finger concurrently. To achieve this feat of multichannel sensorimotor integration, an ANN for efferent control was developed for EMG pattern recognition with each subject. Next, subjects were trained to interpret the haptic feedback sensations from each fingertip individually, and then simultaneously. Finally, the multitasking experiments with the entire robotic system were performed.

#### 1) Training for Simultaneous EMG Slip Control

**Fig. 5.**
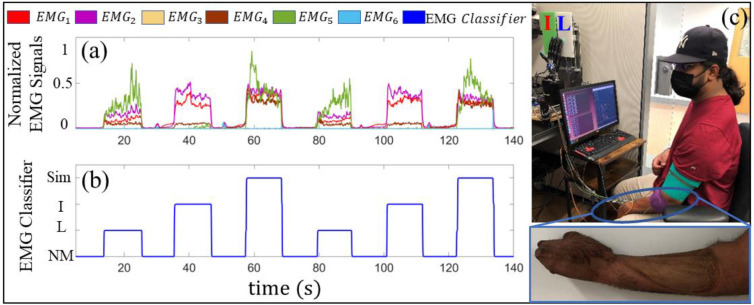
Training subjects for efferent control. (a) Normalized EMG signals from six electrodes. (b) ANN classifier outputs for the Sim, I, L, and NM classes corresponding to the six EMG signals. (c) Subject S3 performing the EMG classifier training. The subject gave permission for the use of his image.

Six EMG signals were recorded from the forearms of each human subject (four electrodes are 13E200 AC, Otto Bock, and 2 electrodes are from Myolab II (Motion Control, Inc., Salt Lake City, USA)). The six electrodes were arranged circumferentially around the forearm with an approximately equal distance between each electrode (Fig. 5(c)). The subjects were asked to flex muscles in their forearms to ensure that all signals responded well and to fine-tune electrode locations and gains.

Next, ANN classifiers were trained for each subject with six EMG inputs and four output classes. The outputs from the ANN classifier were No Motion (NM), Index (I) finger flex, Little (L) finger flex, and Simultaneous (Sim) finger flexion of both index and little fingers (Fig. 5(a, b)). For each class, 10 trials were collected in which the human subjects were asked to follow a 0.1 Hz rectangular pattern with 50% duty cycle generated in Simulink that was displayed on the monitor in front of them (Fig. 5(c)). Time domain data were amplified and then lowpass filtered before using it to train the subject-specific ANN classifiers for EMG pattern recognition. EMG data were separated into four different matrices corresponding to each class and were labeled to train the ANN classifier offline using the same methods previously described for the tactile slip sensation classification problem. After the ANN was trained for each subject, the utility of the classifier was quickly verified in real time in Simulink by asking every subject to produce each EMG class several times before the robotic experiments took place (Fig. 5(c)).

#### 2) EMG Control Algorithms for Simultaneous Slip Prevention

EMG signal processing to control slip simultaneously or individually with two fingers independently or concurrently was accomplished first by normalizing the EMG signals from each electrode. Next, the desired force $\left(F_{D}\right)$ for the index (I) or little (L) finger (Ø∈I,L) was specified as:

(1) $$F_{D,Ø}=\beta_{Ø}\sigma_{Ø}E_{Ø},$$

Where, $\beta_{Ø}$ was the desired force gain for finger Ø. The $\sigma_{Ø}$ term was specified by a look-up table that was based on the EMG class (TABLE I) to determine which finger(s) the subject desired to control. $E_{Ø}$ was the mean of the normalized voltages from the six EMG electrodes.

**Fig. 6.**
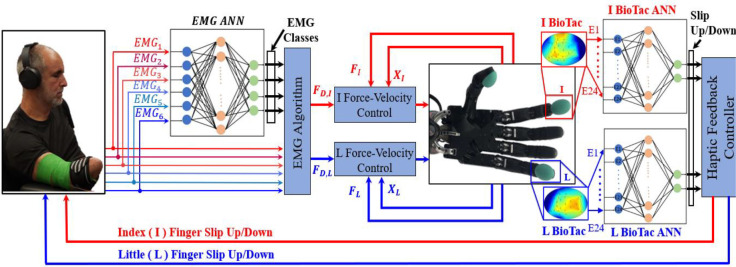
Control system for multichannel sensorimotor integration. Six EMG signals were classified by subject specific ANNs to specify which finger(s) the subjects wanted to control. The EMG signals were also used to specify the desired forces for the index (I) and little (L) fingers that were realized by hybrid force-velocity controllers. ANNs were used with the BioTac SPs on the I and L fingers to classify the 24 taxels at each fingertip into sensations of sliding contact, either up or down. These sensations of touch from each fingertip were encoded via the frequency of vibration and fed back to the subjects with the haptic armband. The subject (S1) gave permission for the use of his image.

The two desired forces ${(F}_{D,Ø})$ were inputs to two hybrid force-velocity controllers [45] that were implemented in MATLAB/Simulink to control the I and L fingers (Fig. 6). The two different controllers for each finger had an outer force feedback loop and an inner velocity feedback control loop. The two hybrid force-velocity controllers for the I and L fingers were designed as described in our prior work [43]. Summarizing, the outer force feedback loop for each fingertip used the corresponding BioTac P_DC_ value to control the applied fingertip force and the measured joint angles ($X_{Ø}$) to control the velocity.

#### 3) Learning Context-Dependent Multi-Digit Haptic Feedback

Training the subjects to interpret the context-dependent haptic feedback from the vibrotactile stimulators was the next step. For the congenitally limb-absent subject S2 and the nine non-amputee subjects S4-S12, the vibrotactile stimulators were placed ipsilaterally on the upper arm over the biceps and triceps for haptic feedback corresponding to the I and L fingers, respectively. However, the amputee subjects S1 and S3 had poor sensitivity in several locations on their upper arms due to scar tissue and requested different haptic feedback locations. After a brief searching process, suitable haptic feedback sites were identified nearer the deltoid and elbow.

The haptic feedback training process started by demonstrating the Slow and Fast vibrotactile stimulator activation modes through Simulink in real time. These two frequencies of activation produced different vibratory sensations (Fig. 4). For example, the amplitude of the steady state pressure (P_DC_) at the Fast frequency (Fig. 4(f)) was approximately half that of the Slow activation frequency (Fig. 4(b). The power spectral distribution of each vibration mode was quite different for both P_DC_ (Fig. 4(c, g)) and P_AC_ (Fig. 4(e, i)), which also helped produce distinguishable haptic signatures for each vibration mode.

Next, the subjects were informed the correlation between the Fast/Slow vibration modes and the direction of slip at the fingertips. Specifically, the Slow vibration mode indicated upward slip, while the Fast vibration mode indicated downward slip. After describing this mapping to the human subjects, their haptic perception was systematically tested. A pseudo-random sequence of 25 trials were generated using the MATLAB randperm command to test their ability to simultaneously detect the directions of slip from both vibrotactile stimulators at the same time. There were five repetitions of each of the five possible combinations of simultaneous vibration activation modes (TABLE 2). All subjects were able to correctly interpret the simultaneous vibrotactile activation modes with 100% accuracy due to the significantly different sensations produced by the Slow and Fast frequencies of actuation (Fig. 4).

#### 4) Training for Multichannel Sensorimotor Integration

Next, the four different combinations of simultaneous slip up/down with the I/L fingers were demonstrated several times for the subjects with the robotic system that was under EMG control. Subjects were allowed approximately five minutes to gain familiarity with multichannel sensorimotor integration using the robotic system to learn how the EMG control signals would stop slip of the two objects individually or simultaneously when desired. After this initial phase, an opaque blockade was placed between the Shadow Hand and the subjects to prevent them from seeing the robotic system, forcing them to rely purely on haptic feedback. The human subjects were also asked to wear noise cancelling headphones (Fig. 6).

During the experiment, the different combinations of simultaneous slip modes were tested with a pseudo-random sequence of 50 trials (10 repetitions for each of the five options listed in TABLE 2, including the control option where haptic feedback was disabled). All the human subjects went through the same pseudo-random sequence that was generated with the MATLAB randperm command. The subjects controlled the pace of the experiments by pressing the Enter key on the keyboard in front of them to initiate each trial. Every trial was designed to be 15 s long, during which, subjects were asked to identify the vibrotactile stimulator activation modes and to maintain the correct EMG class to prevent downward slip. They were trained to permit upward slip at the finger(s) where they perceived upward slip (TABLE 2).

**Fig. 7.**
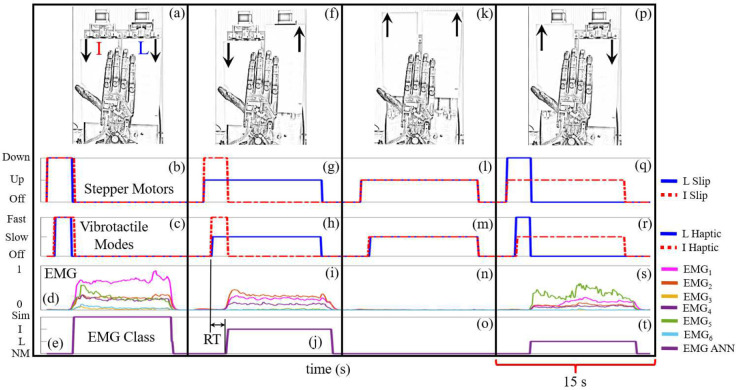
Sample data illustrating robotic system operation for the four cases of simultaneous slip at the index (I) and little (L) fingers. Simultaneous haptic feedback had two different vibration frequencies depending upon the direction of sliding contact. Upward slip was encoded with Slow vibration while the downward slip produced Fast vibration. Subjects were trained to permit upward slip but prevent downward slip using their six EMG signals to produce four different classes (Sim, I, L, NM) with the efferent ANN. (a) Simultaneous downward slip at each fingertip was created by (b) both stepper motors driving downward slip. (c) This caused both vibrotactile actuators to be activated in the Fast mode. (d) The subject perceived the simultaneously activated channels of haptic feedback and increased his EMG signals (e) to produce the Sim class with the efferent ANN. (f, g) Slip down at the I finger with slip up at the L finger caused the (h) vibrotactile stimulators to be actuated with the Fast and Slow modes, respectively. (i) The subject responded to this multichannel haptic feedback to increase his EMG signals and (j) produced the I class. (k, l) Simultaneous upward slip at each fingertip (m) caused both vibrotactile stimulators to be actuated with the Slow mode. (n) The subject did not increase his EMG signals since he desired slip to occur at both fingertips in this case, (o) producing the NM class. (p, q) Slip up at the I finger but slip down at the L finger (r) produced the Slow and Fast vibrational frequencies for the I and L haptic feedback, respectively. (s) The subject responded to this haptic feedback by increasing his EMG signals to (t) produce the L class. The response time (RT) is calculated as the time difference between the onset of haptic feedback and when the subject produced an EMG class (h,j).

The efferent ANN classification accuracy was calculated as the percentage of time that the subjects maintained the correct EMG class during each 15s trial after they responded to the simultaneous slip haptic feedback stimuli. In this scenario, the EMG classification accuracy is also a metric to describe the extent to which subjects were able to integrate the two channels of nuanced haptic feedback into their motor control strategies. Additionally, the response time (RT) was tabulated for each subject and class as the amount of time each person required to interpret the haptic feedback and respond, in other words, the time difference between onset of haptic feedback and activation of an EMG class (Fig. 7(h, j)). The human reaction time to haptic stimuli has often been used as a metric of the cognitive load to operate a prosthetic hand [46, 47].

For both dependent variables (EMG classification accuracy, RT), two factor ANOVA were performed in MATLAB with human subject and EMG class as the two independent variables. Interaction between the human subjects and EMG class was also tested for statistically significant impact upon the classification accuracy and RT. An unbalanced ANOVA was also performed to determine if there was a statistically significant difference between amputee and non-amputee subjects for both dependent variables.

## III. RESULTS

Sample data show the four possible cases of simultaneous slip at the I and L fingers (Fig. 7). When both stepper motors drove the slip platforms downward (Fig. 7(a, b)) the afferent ANNs correctly classified the sensations and the vibrotactile actuators corresponding to the I and L fingers were both activated in the Fast frequency (Fig. 7(c)). This prompted the subject to increase his EMG signals (Fig. 7(d)) to produce the Sim class (Fig. 7(e)) using the efferent ANN, which caused both the slip (Fig. 7(b)) and haptic feedback to cease (Fig. 7(c)). When the sliding contact at the I finger was downward while slip at the L finger was concurrently upward (Fig. 7(f, g)) the vibrotactile actuator corresponding to the I finger was actuated in the fast mode while the corresponding haptic feedback from the L finger was actuated simultaneously in the slow mode (Fig. 7(h)). This caused the subject to increase his EMG signals (Fig. 7(i)) to produce the I class using the EMG ANN (Fig. 7(j)) which halted slip at the I finger (Fig. 7(g)) and the corresponding haptic feedback from the I finger; the haptic feedback from the L finger remained on in the Slow mode since the user desired to permit slip at the L finger only (Fig. 7(h)). When slip at both fingertips was upward (Fig. 7(k, l)), both vibrotactile stimulators were actuated in the Slow mode (Fig. 7(m)) and the subject chose to take no action (Fig. 7(n)) which produced the NM class (Fig. 7(o)) to permit slip at both fingers in this situation (Fig. 7(k, l)). When slip at the I finger was upward while slip at the L finger was simultaneously downward (Fig. 7(p,q)), the afferent ANNs classified this correctly and triggered the corresponding vibrotactile stimulators to be actuated in the Slow and Fast modes to supply haptic feedback from the I and L fingers, respectively (Fig. 7(r)). This prompted the subject to increase his EMG signals (Fig. 7(s)) to produce the L class (Fig. 7(t)) which halted slip at the L finger only (Fig. 7(q, r)).

### A. EMG Classification Accuracy During Multichannel Sensorimotor Integration

**Fig. 8.**
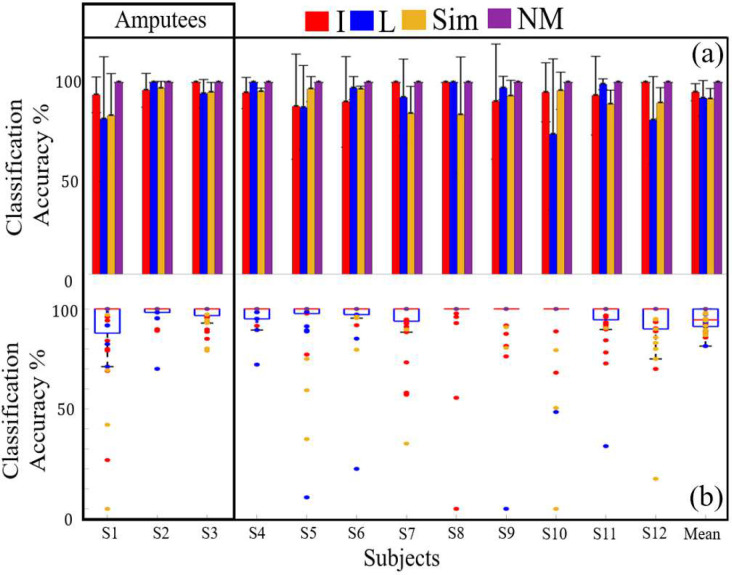
(a) EMG ANN classification accuracy for all subjects with each class demonstrated high proficiency for sensorimotor integration. (b) Box plots showing classification accuracy for each trial with all the subjects.

All participants achieved reasonably high EMG classification accuracy with an overall mean classification accuracy of 95.53% ± 0.23% (Fig. 8), which demonstrated high proficiency for sensorimotor integration during the simultaneous slip control problem. The subject with the highest classification accuracy was S4 with mean accuracy of 97.95% ± 8.15%, while the subject with the lowest classification accuracy was S1 with mean accuracy 91.24%±20.10%. Averaged across all 12 subjects, the highest classification accuracy within an EMG class was the NM class with 100% mean accuracy while the classification accuracy for the I, L and Sim classes was 94.93% ± 4.43%, 91.72%±9.27%, and 91.30% ±5.61%, respectively.

The ANOVA showed that there was no significant difference between the subjects’ EMG classification accuracy or the different classes (p>0.05). However, the interaction between subjects and the classes was significant (p<0.05). An example of this interaction can be seen with subject S8 who had lower accuracy with the Sim class while subject S10 had lower accuracy with the L class.

The mean EMG classification accuracy for amputees was 94.90% ± 13.88%, while the mean EMG classification accuracy for non-amputees was 94.36% ± 15.88%. The unbalanced ANOVA showed that the amputees’ EMG classification accuracy was not significantly different from the non-amputees (p>0.05).

### B. Subjects’ Response Time to Multichannel Haptic Feedback

**Fig. 9.**
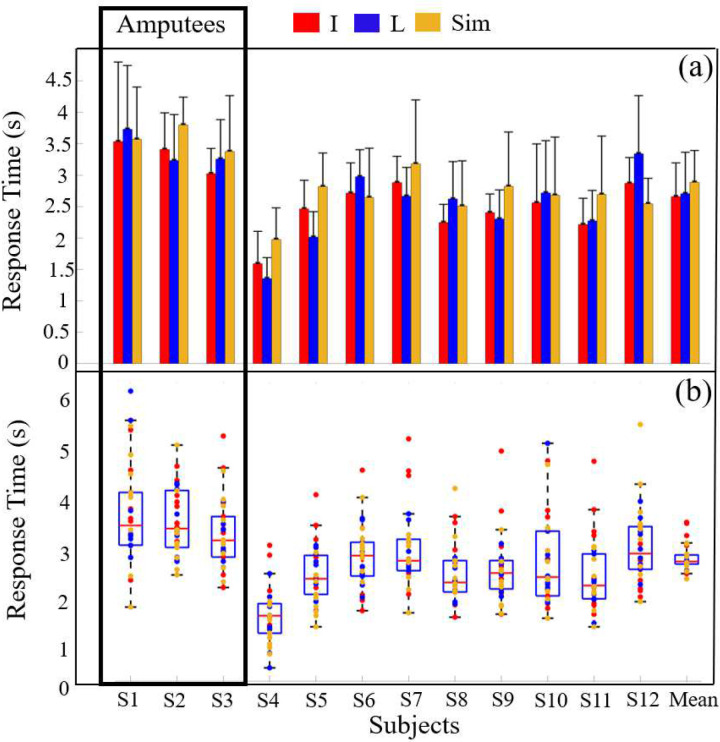
(a) Each subject’s response time for the I, L, and Sim classes. (b) Box plots for every subject’s response times with each class. The amputees had a significantly longer time to respond to the haptic stimuli than the non-amputees.

The mean RT of the three ULA people was 3.45s ± 0.80s, while the mean RT for non-amputee subjects was 2.53s ± 0.76s (Fig. 9). The average RT across all 12 subjects was 2.75s ± 0.54s. Subject S4 had the fastest mean RT of 1.62s ± 0.53s while subject S1 had the slowest RT of 3.59s ± 0.94s. The three amputee subjects (S1-S3) had the three slowest average RTs within the cohort.

The ANOVA on the RT across all subjects showed a statistically significantly difference (p<0.01). The RT for each class was also statistically significant (p<0.01). However, the interaction between the subject’s performance and the classes was not statistically significant (p>0.05). The unbalanced ANOVA showed that the amputee RT was significantly different than the non-amputee RT (p<0.01).

## IV. Discussion

It remains an open question on the best approaches to maximize the human-prosthesis bandwidth for bidirectional information transfer. EMG pattern recognition for efferent control signals has shown a great deal of interest in recent years. Building off this prior research, several commercially available products have emerged. Otto Bock has the Myo Plus pattern recognition system that relies upon eight EMG electrodes to classify the intended grip pattern of the amputee from forearm muscle signals [48]. Complete Control is a related product offered by Coapt for EMG pattern recognition and control of multiple DOFs of a prosthetic arm [49].

In contrast, most prosthetic hands still do not have tactile sensors [50] despite the significant amount of research on sensors and signal processing [51]. Consequently, haptic feedback for ULA people in clinical practice is largely unrealized. The explanation for this dearth of haptic feedback is multifaceted. The strict size, mass and power constraints on prosthetic limbs impose a difficult barrier to overcome, where performance tradeoffs must always be made. There are also the questions of the most important modalities of haptic feedback [25, 52], whether to encode tactile events spatially or temporally [53], and the number of independent haptic feedback channels required for satisfactory control [10].

Expanding upon the question of the required number of haptic feedback channels, one reasonable viewpoint is that the capacity for sensation should be commensurate with the capability for control. Clearly, this has yet to come to fruition in terms of commercial products available to amputees—efferent control advancements have outpaced afferent feedback products. However, a different viewpoint is that the richness of haptic feedback should exceed the capability for control. There is a strong physiological precedent for this notion as the quantity of mechanoreceptors per fingertip number in the thousands [41]. We have explored the latter viewpoint in this paper, where the sensation space was larger than the action space both at the robotic level and from the perspective of the human in the loop. At the robotic level, we used ANNs for 24 taxels per fingertip to produce two haptic feedback sensations corresponding to upward or downward sliding contact. And for the human in the loop, there were five possible combinations of sensations with only four possible control classes (TABLE 2) because of the situations where the same control choice would be a desirable response to different sensations. Enhancement of artificial hand sensation and haptic feedback will be fruitful avenues of future investigation to improve prosthetic arm embodiment [54] and reduce the prevalence of artificial limb rejection [55], which remains high because of poor dexterity and lack of sensory feedback, among other reasons [56].

### A. Cognitive Load and Response Time to Haptic Stimuli

The impact of haptic feedback upon the cognitive load to operate a prosthetic hand has often been quantified by the human reaction time to haptic stimuli [46, 47]. For example, it has been shown that amplitude modulation from a single vibrotactile stimulator can help reduce reaction time and cognitive load compared to spatially encoding the feedback via multiple stimulators [53]. It is well known that haptic feedback from a single channel can help reduce the cognitive load to perform a single task; however, the cognitive load will increase when scaling up the number of feedback channels [10]. Nevertheless, dexterous prosthetic hands of the future will require more sensory feedback to improve their functionality. In our paper, we used two channels of haptic feedback that were simultaneously activated with frequencies dependent upon the direction of sliding contact. The ANOVA showed a statistically significant impact of the simultaneous vibration mode upon the RT (p < 0.05; Fig. 9). In particular, the Sim class had the slowest RT averaged across all subjects and this is the situation where both vibrotactile stimulators were activated with the same Fast frequency. The other situations requiring an action from the subjects (I, L classes in Fig. 9) had both the Slow and Fast frequencies of vibration simultaneously from the two stimulators, and so could have been easier for most subjects to discern more rapidly. Subjects S6 and S12 were the only two people who had the fastest mean responses with the Sim class; the other ten subjects responded more quickly on average in situations where the frequencies of vibration from the two stimulators were different (I,L classes).

Regarding real-time efferent control, EMG pattern recognition approaches can reduce the cognitive load required to operate myoelectric hands [57], but there are some differing reports regarding the utility of this approach for ULA people relative to non-amputees. For example, a comparison between two amputees and 12 non-amputees showed that amputees had a lower success rate and required more time to complete a pick and place task with different objects using a regularized discriminant analysis grip classification algorithm and a hybrid EMG-inertial measurement unit sensor suite [58]. In contrast, another study used a KNN algorithm for EMG pattern recognition in which no statistically significant difference was found between non-amputees and amputees for the time needed to select a desired motion of myoelectric prostheses [23]. But as is noted in the aforementioned studies, additional experiments should be done with a larger sample size to explore the impact of congenital or traumatic hand amputation upon cognitive load required to operate dexterous prostheses. It is possible that with additional training time [59], differences that may exist between amputees and non-amputees could be mitigated with use of supporting apps to enable in-home practice and virtual visitations with clinicians. The ultimate goal should be to maximize prosthetic hand dexterity and functionality while minimizing cognitive load required for operation.

The data in our paper support the idea that amputees may have a higher cognitive load required in sensorimotor integration tasks involving pattern recognition of multiple channels of efferent and afferent information in comparison to non-amputees. This is supported by the fact that the three amputees in our study had a significantly slower RT to perform the multitasking experiments in comparison to the nine non-amputees (Fig. 9; p<0.01); however, the amputees’ EMG classification accuracies were not significantly different than the non-amputees (Fig. 8; p>0.05). A traumatic upper limb amputation drives a large scale cortical reorganization at the network level, extending beyond the sensorimotor cortex [60], which could have affected the RT of the ULA subjects S1 and S3 who suffered amputations as adults. However, subject S2 who has a congenital limb deficiency, also had a significantly slower RT. During development, children with congenital limb deficiencies are deprived of afferent peripheral sensations that would normally shape the function of the assumed hand territory of the brain. Instead, compensatory movements involving the feet, lips, and forearm may provide afferent inputs to this region of the brain during maturation [61]. It therefore stands to reason that providing haptic feedback to children who are born without a hand could be highly beneficial to their future potential as adults.

## V. Conclusion

The data in our paper have shown that people can interpret context-dependent information from two different vibrotactile stimulators simultaneously to concurrently control individual fingers of a dexterous artificial hand with EMG pattern recognition using an ANN (Fig. 8). We explored the potential of three amputees and nine non-amputees to perform this complex feat of multichannel sensorimotor integration in the context of deciding whether to permit or prevent sliding contact at two fingertips of an artificial hand individually and simultaneously. This kind of situational awareness underpins refined manual dexterity inherently required to perform sophisticated tasks such as playing sports or a musical instrument like a violin (Fig. 1) [3]. Our results showed that the subjects were able to correctly interpret the vibrotactile feedback from both haptic feedback channels simultaneously to correctly and concurrently control two fingers of the artificial hand with 95.53% ± 0.23% accuracy averaged across all 12 subjects. There was no statistically significant difference in the classification accuracy between the amputees and non-amputees; however, the amputees did require significantly more time to respond to the haptic stimuli in comparison to the non-amputees. This suggests that the amputees experienced a higher cognitive load to perform the same sensorimotor integration task that required interpretation of two channels of haptic feedback simultaneously. These results provide a small step towards the grand challenge of enabling amputees to dexterously control prosthetic hands.

## Figures and Tables

**TABLE I:** Correlation between EMG class from the efferent ANN and the desired force (*F*_*D*,Ø_) for each finger Ø ∈ I, L.

EMG Class	Index Finger (*σ_I_*)	Little Finger (*σ_L_*)
NM	0	0
*Sim*	1	1
*L*	0	1
*I*	1	0

**TABLE 2:** Sensation and action scenarios during the simultaneous slip experiments that required multichannel sensorimotor integration with the index (I) and little (L) fingers.

Haptic Feedback		Slip Sensation		Actions		Correct EMG Class
I	L	I	L	I	L	
Slow	Slow	↑	↑	x	x	NM
Fast	Fast	↑	↓	√	√	Sim
Slow	Fast	↑	↓	x	√	L
Fast	Slow	↓	↑	√	x	I
Off	Off	None	None	x	x	NM

## Data Availability

The data that support the findings of this study are available upon request.

## List of abbreviations

ANN: Artificial Neural Network
EMG: Electromyogram
SIM: SIMULTANEOUS
I: Index finger
L: LITTLE FINGER
NM: No motion
ULA: Upper Limb Absent
